# Legislation restricting gender-affirming care for transgender youth: Politics eclipse healthcare

**DOI:** 10.1016/j.xcrm.2022.100719

**Published:** 2022-08-16

**Authors:** Katherine L. Kraschel, Alexander Chen, Jack L. Turban, I. Glenn Cohen

**Affiliations:** 1 Solomon Center for Health Law & Policy, Yale Law School, New Haven, CT, USA; 2 Harvard Law School, Cambridge, MA, USA; 3 Division of Child & Adolescent Psychiatry, Stanford University School of Medicine, Stanford, CA, USA

## Abstract

In the past two years, in 25 US states, bills have been introduced to restrict access to gender-affirming medical care for minors. Some have already become law. We show how these bills, while purporting to “protect” trans youth, are really an assault on their ability, along with their parents’ and physicians’, to make healthcare choices and to receive medically necessary care. We discuss the evidence-based guidelines for the care of these patients, the positions taken by major medical societies against these bills, and the landscape of legal challenges that are being brought against these enacted laws.

## Teaser

In the past two years, in 25 US states, bills have been introduced to restrict access to gender-affirming medical care for minors. Some have already become law. We show how these bills, while purporting to “protect” trans youth, are really an assault on their ability, along with their parents’ and physicians’, to make healthcare choices and to receive medically necessary care. We discuss the evidence-based guidelines for the care of these patients, the positions taken by major medical societies against these bills, and the landscape of legal challenges that are being brought against the enacted laws.

## Main text

Legislators in 25 US states have introduced bills to restrict access to gender-affirming medical care for minors in the past two years (Table 1). To date, these bills have become law in Alabama, Arkansas, and Arizona. On their face, these efforts claim to “protect” trans (we use the terms “trans” and “transgender” interchangeably) youth. However, as we discuss in this commentary, far from helping trans youth, these laws prevent them from receiving medically necessary care that learned professional societies have established. For this reason, relevant professional organizations including The American Medical Association, The American Academy of Pediatrics, The American Psychiatric Association, and The American Academy of Child & Adolescent Psychiatry have explicitly voiced opposition to these laws.^1^ These proposed bills and laws share common flaws—they are based on false claims about standards of care and health outcomes for people with gender dysphoria, and they are based on inaccurate, biased, and misleading representations of the evidence base.^2^

**Table 1:** Proposed state bills and laws to limit access to gender-affirming medical care for transgender adolescents in 2021 and 2022

State	bill and brief summary of some major provisions
Alabama	HB1/SB10 (2021)/HB266/SB184 (enacted 2022): Criminalizes the provision of some gender-affirming medical or surgical care to transgender adolescents and requires school personnel to reveal the gender identity of transgender youth to their parents.
Arizona	SB1511 (2021): Adds some gender-affirming medical and surgical care to the state’s definition of child abuse and criminalizes physician activity of this sort. SB1138 (enacted 2022): Prohibits the provision of gender-affirming surgical care to transgender minors.
Arkansas	HB1570/SB347 (enacted 2021): Prohibits the provision of some gender-affirming medical or surgical care to transgender adolescents and prohibits the use of public funds for gender-affirming care. Recently became law when the state legislature overrode the Governor’s veto.
Florida	HB935 (2021)/HB211(2022): Criminalizes the provision of some gender-affirming medical and surgical care to transgender adolescents.
Georgia	HB401 (2021): Criminalizes the provision of gender-affirming medical and surgical care to transgender adolescents. Creates civil claim against medical professionals who provide gender-affirming care to transgender minors.
Idaho	HB675 (2022): Criminalizes the provision of gender-affirming medical care to transgender adolescents. Adds gender-affirming medical care to the definition of genital mutilation of a child.
Indiana	SB224 (2021): Prohibits the provision of gender-affirming surgical and medical care to transgender minors.
Iowa	HF193 (2021): Subjects health professionals to civil liability and disciplinary sanction for the provision of some gender-affirming medical and surgical care to transgender adolescents.
Kansas	HB2210 (2021): Criminalizes the provision of some gender-affirming medical or surgical care to transgender adolescents.
Kentucky	HB253/SB84 (2022): Subjects health professionals to civil liability and disciplinary sanction for the provision of some gender-affirming medical and surgical care to transgender adolescents. Prohibits the use of public funds for gender-affirming care to minors.
Louisiana	HB575 (2021)/HB570 (2022): Criminalizes the provision of some gender-affirming medical or surgical care to transgender adolescents. Prohibits school personnel to withhold from parents or legal guardians “information related to a minor’s gender or sex that is inconsistent with the minor’s sex.” Creates civil liability for providers and parents in violation. Prohibits the use of public funds for gender-affirming care to minors.
Mississippi	HB1147 (2022)/SB2728 (2022): Prohibits provision of or referral for gender-affirming surgical and medical care to transgender minors. Prohibits the use of public funds for any gender-affirming medical care to transgender minors.
Missouri	HB33 (2021): Subjects medical professionals who provide some gender-affirming medical or surgical care to transgender adolescents to possibility of healthcare license revocation. Establishes that parents or guardians who obtain some gender-affirming medical or surgical care for transgender adolescents shall be reported to the state’s child welfare division. HB2649 (2022): Subjects medical professionals who provide some gender-affirming medical or surgical care to transgender adolescents to possibility of healthcare license revocation. Disallows public funds to any organization or individual who provides gender-affirming care to transgender minors. Creates civil claim against medical professionals who provide gender-affirming care to transgender minors.
Montana	HB 427 (2021): Prohibits the provision of gender-affirming surgical and medical care to transgender minors.
New Hampshire	HB68 (2021): Criminalizes the provision of gender-affirming healthcare to transgender minors.
North Carolina	SB514 (2021): Subjects medical professionals who provide some gender-affirming medical or surgical care to transgender adolescents to having their healthcare license revoked and authorizes civil liability. Prohibits use of public funds for gender-affirming care. Requires government employees to reveal the gender identity of transgender youth to their parents in writing. Prohibits the use of public funds for gender-affirming medical or surgical care.
Ohio	HB 454 (2021): Prohibits the provision of gender-affirming surgical and medical care to transgender minors. Subjects providers to possibility of healthcare license revocation and civil liability. Prohibits the use of public funds for gender-affirming care to transgender minors.
Oklahoma	SB583 (2021)/HB3240 (2022): Subjects medical professionals who provide some gender-affirming medical or surgical care to transgender adolescents to have their healthcare license revoked. SB676 (2021): Criminalizes the provision of some gender-affirming medical or surgical care to transgender adolescents. Establishes criminal penalties for parents who obtain some gender-affirming medical or surgical care for their children. HB3240 (2022): Creates civil liability for providers and parents in violation. Prohibits the use of public funds for gender-affirming care to minors.
South Carolina	HB4047 (2021): Criminalizes the provision of some gender-affirming medical or surgical care to transgender adolescents. Requires school personnel to reveal the gender identity of transgender youth to their parents.
South Dakota	HB1057 (2020): Criminalizes the provision of gender-affirming medical or surgical care to transgender adolescents.
Tennessee	SB657 (2021): Criminalizes the provision of gender-affirming medical or surgical care to a transgender adolescent unless both parents or guardians of the adolescent provide a signed written statement from two physicians and an additional board-certified child and adolescent psychiatrist recommending such interventions. HB2835/SB2696 (2022): Subjects medical professionals who provide some gender-affirming medical or surgical care to transgender adolescents to have their healthcare license revoked. Creates civil penalty for medical professionals. Prohibits use of public funds by any entity or person providing gender-affirming care to a minor.
Texas	HB68 (2021): Adds some gender-affirming medical and surgical care to the state’s definition of child abuse. HB1339 (2021): Prohibits the provision of some gender-affirming medical or surgical care to transgender adolescents Prohibits malpractice insurance providers from providing coverage for damages related to gender-affirming medical or surgical care for transgender adolescents. Governor’s Executive Directive (2022): Orders investigations into parents and medical facilities providing healthcare to transgender adolescents. Based on non-binding interpretation from attorney general that classified gender-affirming care as child abuse.
Utah	HB127 (2022): Subjects medical professionals who provide some gender-affirming medical or surgical care to transgender adolescents to have their healthcare license revoked.
West Virginia	HB2171 (2021): Criminalizes the provision of some gender-affirming medical or surgical care to transgender adolescents.
Wisconsin	SB915 (2022): Prohibits the provision of gender-affirming surgical and medical care to transgender minors. Creates civil liability for providers in violation. Prohibits the use of public funds for gender-affirming care to minors.

Table adapted from and expanded from the one published in ref.^1^ Table up to date as of July 1, 2022.

The mechanisms by which these laws and proposed legislation seek to limit access to care vary. Some states would criminalize the acts of medical professionals or parents for providing care. For example, Alabama’s law, passed in April of this year, makes providing pubertal suppression or gender-affirming hormones to minors a Class C felony, punishable with up to ten years in prison. Some states would require a medical licensing board to discipline and possibly revoke the license of professionals who provide gender-affirming care to minors. Some states have also considered other modes of restriction, including imposing reporting requirements on educators or limiting public funding.

Thankfully, most of these bills have not passed, and some are no longer under consideration. However, given the growing number of states in which this type of legislation was introduced this year and the number of states that reintroduced legislation in 2022 that failed to pass in 2021, state legislators show no signs of relenting. In addition, if the legislative process fails, some states may take action through their executive branch. The Texas legislature did not pass proposed legislation that would have stripped Texas healthcare providers of their medical license for providing gender-affirming care to minors and made such care child abuse. In response, Governor Greg Abbott issued an Executive Directive, affirming a non-binding opinion of the state attorney general that gender-affirming care constitutes child abuse and ordering its Department of Family and Protective Services to investigate parents who provide their children such care. As discussed below, enforcement of the Alabama and Arkansas laws is currently blocked by federal courts while legal challenges proceed, and Governor Abbott’s order is enjoined by Texas state courts.

### Standards of care in treating transgender youth

There is nothing inherently unhealthy or abnormal about being trans. However, some trans individuals suffer from a condition termed gender dysphoria, the criteria for which are set out in the American Psychiatric Association’s Diagnostic and Statistical Manual for Mental Disorders (DSM) and include “a marked incongruence between one’s experienced/expressed gender and assigned gender, of at least six months’ duration, that is associated with clinically significant distress or impairment in social, occupational, or other important areas of functioning.”^3^

A series of evidence-based clinical guidelines set out the treatment for gender dysphoria, in particular the Endocrine Society Clinical Practice Guideline for Endocrine Treatment of gender-dysphoric/gender-incongruent persons and the World Professional Association for Transgender Health Standards of Care for the Health of Transsexual, Transgender, and Gender-Nonconforming People.^4^^,^^5^ These guidelines set out the criteria for who is competent to provide gender-affirming care as well as the comprehensive processes that should be followed prior to initiating care. They emphasize that medical interventions are not considered for prepubertal children and that interventions for adolescents are considered in a stepwise fashion from most reversible (i.e., pubertal suppression) to less reversible (i.e., gender-affirming hormones including estrogen or testosterone).

While some of these interventions are reversible (i.e., the temporary pausing of endogenous puberty from gonadotropin-releasing hormone agonists [GnRHas]), endogenous puberty itself is irreversible and can cause substantial lifelong psychological distress for those with gender dysphoria, while also often creating the need for more-invasive surgeries later in life. In addition to their use for gender dysphoria, GnRHas have been used in the treatment of central precocious puberty dating back to the 1970s,^4^ providing longitudinal safety data for their use in the pediatric population. Research from the precocious puberty literature has shown that, despite assertions by some legislators, these medications do not appear to cause infertility.^6^ However, there is some concern that going directly from pubertal suppression to gender-affirming hormones like estrogen or testosterone may impair fertility. For that reason, existing guidelines recommend fertility counseling prior to adolescent patients pursuing such care, so that they may consider fertility-preservation options.^4^

Under existing guidelines, gender-affirming genital surgery is not considered for minors, but gender-affirming chest surgery may be considered for trans masculine adolescents on a case-by-case basis, weighing the substantial risks of surgery against the potential mental and physical health benefits for each individual patient.^7^^,^^8^ Current guidelines highlight the importance of both informed consent from a minor’s parents and informed assent from the minors themselves prior to the initiation of any gender-affirming medical or surgical care. Alabama’s law provides an example of the flawed reasoning behind these laws. It erroneously claims, among other things, that standard treatment for a transgender adolescent would include genital surgery, when, in fact, the current consensus in the field is to wait until the patient reaches the age of majority before pursuing such surgical procedures.

### Documented benefits of gender-affirming care

A substantial body of literature exists documenting the benefits of gender-affirming medical interventions, where indicated, for adolescents with gender dysphoria. Over a dozen studies have collectively linked such care to improvements in depression, anxiety, and suicidality.^9^^,^^10^ Nonetheless, legislators have ignored or omitted any mention of the benefits of gender-affirming care in their legislative findings and have overstated the number of adolescents whose gender dysphoria dissipates without gender-affirming care.

It is problematic for the state to interpose itself and prevent the provision of care that is evidence-based, meets clinical guidelines, and takes place in circumstances where parents, adolescents, and healthcare providers are all aligned and supportive of what they view as the best medical treatment for a given adolescent. Legislators appear to have singled out trans adolescent care while allowing professionals, parents, and adolescents to together decide the best course of action for *other* treatments posing potential risks. For example, states have not sought to prohibit, let alone criminalize, the performance of pediatric breast reduction to address excess breast tissue, back pain, or social anxiety. Indeed, it is telling that a state like Arkansas that bans gender-affirming care expressly allows surgical inventions for minors with intersex conditions, even though such procedures have irreversible, long-term consequences.^11^ Arkansas permits these procedures with infants that are too young to consent, yet it prohibits gender-affirming care when competent adolescents agree with their parents and healthcare providers that such standard of care treatment is in the patient’s best interest. Many of these laws prohibit the use of GnRHas for gender dysphoria while still allowing use of these medications for central precocious puberty. All this is even more startling against the backdrop of the practice of medicine outside care for trans patients—many standard-of-care treatments carry some risk to the patient, but the net risk-to-benefit ratio combined with the patient’s consent justifies going forward. A rule that requires an intervention to be absolutely free of risk would rule out much of current medical practice, yet that is what legislators are selectively applying to gender-affirming care.

Healthcare providers are fiduciaries for their patients. They are trained, guided by practice guidelines, and use their discretion and medical judgment in partnership with their patient (and in the case of a minor, the patient’s parents as well) to provide care that is in their patient’s best interests. These statutes would transform their fiduciary duty into a criminal act. In many states, criminal prosecutions begin with “The People v.,” reflecting the idea that criminalization is a way a community communicates its moral opprobrium. The criminalization of medical care, with its attendant chilling effect, should be avoided in all but the clearest cases of misconduct. Far from misconduct, the care these providers seek to give assenting adolescents and their consenting parents is evidence-based and guided by established guidelines of the profession. As has always been true in areas like medical malpractice, in determining what is the standard of care, courts and legislators should look to the medical profession as reflected by the leading medical bodies to determine the best medical practices for patients. Once again, gender-affirming care is being singled out in a way one would not countenance for other areas of medicine.

In addition to their flawed reasoning, suspect justifications, and problematic intrusion into the practice of medicine, laws restricting transgender minors’ access to gender-affirming care also raise legal issues. They may violate the US Constitution, state constitutions, the Affordable Care Act, or the Americans with Disabilities Act.

Transgender minors, their parents, and their healthcare providers have brought several legal challenges against restrictions to accessing gender-affirming care for transgender youth.^12^, ^13^, ^14^ Preliminary rulings in Alabama and Arkansas indicate that these laws face significant legal headwinds because of the ways they infringe on the rights of transgender minors, their parents, and their providers as protected by the US Constitution’s 14th Amendment Equal Protection and Due Process Clauses.

First, these laws violate the rights of transgender minors under the 14th Amendment’s Equal Protection Clause because they constitute sex-based classifications that discriminate against transgender people without an “exceedingly persuasive” justification. In the Alabama case, a federal court rejected Alabama’s argument that its criminal ban was constitutional because it protected children against “experimental” treatments, finding that Alabama “produce[d] no credible evidence to show that transitioning medications are ‘experimental,’” and that “at least twenty-two major medical associations in the United States endorse these medications as well-established, evidence-based methods for treating gender dysphoria in minors.”^12^ And in the Arkansas case, a federal court held that Arkansas’ stated rationale of protecting children was pretextual because its law allowed the same types of treatments for cisgender minors that it banned for transgender minors.^13^ The Alabama case also similarly found that “medical providers have used transitioning medications for decades to treat medical conditions other than gender dysphoria, such as central precocious puberty… [and] hormone therapies for patients whose natural hormone levels are below normal.”^12^

Second, these laws interfere with the fundamental rights of parents to direct the care, custody, and control of their children under the 14th Amendment’s Due Process Clause. Courts have recognized that this right includes “the fundamental right to seek medical care for their children and, in conjunction with their adolescent child’s consent and their doctor’s recommendation, make a judgment that medical care is necessary,”^13^ which includes “transitioning medications subject to medically accepted standards.”^12^

Third, these laws likely violate the Equal Protection rights of physicians who provide gender-affirming care to transgender patients by treating them worse than physicians who provide other types of medically accepted care.^13^ In the Arkansas case, the court expressed grave concern that Arkansas’ law “interfer[es] with the patient-physician relationship, unnecessarily regulat[es] the evidence-based practice of medicine and subject[s] physicians who deliver safe, legal, and medically necessary care to civil liability and loss of licensing.” By barring “healthcare providers in [Arkansas from] consider[ing] the recognized standard of care for adolescent gender dysphoria,… the State has ensured that its healthcare providers do not have the ability to abide by their ethical standards, which may include medically necessary transition-related care for improving the physical and mental health of their transgender patients.”^13^

Finally, as is the case in Texas, if legislatures decline to pass restrictions and a state’s executive branch responds by issuing declarations or orders to restrict or criminalize care, such executive action may be vulnerable to legal challenges under states’ administrative procedures acts. In Texas, a state court issued a temporary restraining order prohibiting the Department of Family and Protective Services from following Governor Abbott’s directive to investigate parents of transgender youth accessing gender-affirming care. The court issued the order, concluding that the plaintiffs stated a valid cause of action that Governor Abbott violated Texas’s Administrative Procedures Act.^14^ Other challenges may also be possible, including claims under state constitutions and federal enforcement of the Affordable Care Act’s anti-discrimination provision or the Americans with Disabilities Act.

In short, lower courts are likely to continue to rule that “[p]arents, pediatricians, and psychologists—not the State or [a] Court—are best qualified to determine whether transitioning medications are in a child’s best interest on a case-by-case basis.”^12^ Although not binding in the United States and not involving *parental* consent, the United Kingdom’s Court of Appeal took a significant position in Bell v. Tavistock, overturning a lower court ruling that severely curtailed the administration of puberty-suppressing medications to transgender minors and was based on the proposition that minors are highly unlikely to have the ability to consent to such treatments.^15^ The Court of Appeal ruled that doctors, rather than a blanket legislative or judicial rule, should determine on a case-by-case basis whether minors are able to consent to any specific treatment.^15^

It is less clear whether courts will be prepared to strike down bans on gender-affirming surgeries for transgender minors—in the Alabama case, the court declined to enjoin a provision of the Alabama law that “bans sex-altering surgeries on minors” without legal analysis.^12^

### Conclusion

Cynics will see the more than 25 bills seeking to interfere with the gender-affirming care for trans youth as just one more tried-and-true attempt to use the lives and freedoms of sexual and gender minority Americans for political advantage as election season looms. We have argued that these bills ignore the evidence-based clinical guidelines that set out the treatment for gender dysphoria and the evidence base documenting the benefits of gender-affirming medical interventions, where indicated, for adolescents with documented gender dysphoria. We also highlight how it is ethically problematic for the state to interpose itself into the individual medical decisions of adolescents, their parents, and healthcare providers by preventing the provision of evidence-based care that meets clinical guidelines. These laws may disproportionally affect some of the most marginalized within the trans community, those without the resources or support to move or travel out of state for care. As we discuss, some of these laws have already faced legal challenges on a myriad of theories. Those fighting for trans youth to have the freedom to choose gender-affirming care have succeeded in some of the court challenges thus far. It is not yet clear how these challenges will culminate given a conservative Supreme Court that has increasingly deferred to states and expressed skepticism of constitutional rights to make very personal medical choices, as we have recently seen with abortion.

While this commentary has focused on the US where these legislative attacks have intensified over the last two years, we are also seeing attacks on the rights of trans youth across the globe. As a small and insular minority, we are sad to see trans youth become targets for political gain.
